# Anmerkungen zum Terminus „Drainage“

**DOI:** 10.1007/s00106-022-01242-1

**Published:** 2022-11-08

**Authors:** Arne Böttcher, Simon Meier-Vieracker

**Affiliations:** 1 Klinik und Poliklinik für Hals‑, Nasen- und Ohrenheilkunde, Kopf- und Neurozentrum, Universitätsklinikum Hamburg-Eppendorf, Martinistraße 52, 20246 Hamburg, Deutschland; 2 Institut für Germanistik, Technische Universität Dresden, Dresden, Deutschland

**Keywords:** Nomenklatur, Drain, Redon, Paukenröhrchen, Paukendrainage, Nomenclature, Drain, Redon, Ventilation tube, Grommet

## Abstract

**Hintergrund:**

Der Gebrauch des Terminus „Drainage“ ist im klinischen Alltag und in der existierenden Fachliteratur inkonsistent.

**Ziel der Arbeit:**

Ziel waren die Klärung der Bedeutung des Terminus und die Sensibilisierung zum fachgerechten Gebrauch.

**Material und Methoden:**

Dazu erfolgte eine selektive Literaturrecherche.

**Ergebnisse:**

Eine Drainage, entlehnt aus dem Englischen, ist der Vorgang des Flüssigkeitsentziehens, wird jedoch häufig mit dem oftmals zeitgleich eingelegten Drainrohr synonym verwendet, was sprachwissenschaftlich als Metonymie bezeichnet wird. Ein Paukenröhrchen dient der Mittelohrbelüftung und dem Druckausgleich. Lymph- und Abszessdrainagen sind keine Gegenstände.

**Schlussfolgerung:**

Die normierte Fachterminologie dient dazu, Unschärfen und konsekutiv Missverständnisse im wissenschaftlichen Kontext zu verhindern. Dies ist besonders in der Medizin von großer Bedeutung, jedoch im alltäglichen Sprachgebrauch einer gewissen Variabilität unterzogen.

## Abstract (en)

**Background:**

Use of the term “drainage” is inconsistent in clinical practice and the existing literature.

**Objective:**

The aim of this study was to clarify the meaning of the term and to raise awareness of its proper use.

**Materials and methods:**

A selective literature search was performed.

**Results:**

Drainage is the process of removing fluid but the term is often used interchangeably with the drain tube, which is often inserted at the same time, which is linguistically referred to as a metonymy. A tympanostomy tube is used for middle ear ventilation and pressure equalization. Lymphatic and abscess drainage are not objects.

**Conclusion:**

Standardized technical terminology serves to prevent vagueness and consecutive misunderstandings in a scientific context. This is particularly important in medicine, but is subject to a certain variability in everyday language use.

Detaillierte terminologische Differenzierungen sind in Anbetracht eines stetig wachsenden Wissenspools unverzichtbare Grundlage wissenschaftlicher Kommunikation, nicht zuletzt, um Missverständnisse zu verhindern. Dies ist in vielen Fachbereichen, aber natürlich auch in der Medizin, von besonderer Bedeutung, zumal die Terminologie hier einem steten Wandel unterzogen ist [1–3]. Bezüglich der zum Teil inkonsistent gebrauchten Nomenklatur möchten wir an dieser Stelle kurz festhalten, dass es sich bei einer Drainage um den Vorgang des Flüssigkeitsentziehens und nicht um den hierfür genutzten Gegenstand handelt. Das Wort „Drainage“, entlehnt aus dem Französischen, das wiederum aus dem Englischen (drainage) entlehnt wurde und ursprünglich in landwirtschaftlichen Kontexten verwendet wurde [4], bedeutet nach Oxford Dictionary: „the process by which water or liquid waste is drained from an area“ [5]. Ebenso gibt der Duden die Bedeutung als „Ableitung von Wundabsonderungen bzw. Flüssigkeiten [nach außen] mithilfe eines Schlauchs oder Röhrchens“ [6] wieder und fokussiert mithin auch auf den Vorgang. Zudem wird korrekterweise im *Springer Kompaktwörterbuch Medizin* die Drainage als „Ableitung von Flüssigkeiten aus dem Körper“ definiert, wohingegen der Drain das hierbei verwendete „Hilfsmittel [dünner Schlauch, Röhrchen]“ ist [7]. Die gleiche Unterscheidung wird aktuell vom Pschyrembel Online vorgenommen [8] und findet sich bereits in der 31.–34. Auflage des damals noch Dornblüth – Pschyrembel genannten Wörterbuchs von 1939 [9]. Die Systematisierte Nomenklatur der Medizin (SNOMED), für die derzeit noch keine deutsche Übersetzung vorliegt, bezeichnet auf amerikanischem Englisch „drainage“ als eine „Evacuation procedure (procedure)“ [10].

Bereits Herrmann Schwartze schlug 1878 vor, „der Incision des Abscesses sofort die Dilatation der Knochenfistel mit dem Meissel und Drainage des Warzenfortsatzes folgen zu lassen“. Ferner schrieb er, „die Höhle nachträglich gründlich ausgespült und ein Drainrohr eingelegt“ zu haben [11].

Otto Körner beschrieb in einer Kasuistik eines Großhirnabszesses von 1890 das Vorgehen, bei dem „ein Drain eingeführt“ wurde und ein „Drainrohr und Jodoformgaze herausragten.“ In einem weiteren Fall heißt es nomenklatorisch korrekt, dass „keine Drainage des Abscesses“ erfolgte [12]. In *Roth’s klinische Terminologie* von 1914 wird Drainage als „Die Ableitung der Wundsekrete“ bezeichnet, die „durch Einlegen von silbernen oder Kautschukröhrchen (Drain)“ erfolge [13]. Im sog. Vademecum von 1957 ist von „Drainage […] durch a- oder antiseptische Gaze […] oder durch Gummi- oder Glasrohr“ die Rede, was sich somit ebenfalls auf die Prozedur bezieht [14].

Erste terminologische Unschärfen findet man beispielsweise bei Kleinschmidt 1948 in einer Therapiebeschreibung des Retropharyngealabszesses: „Eine Dränage muß wegen der Gefahr der Mischinfektion möglichst vermieden werden“ [15]. Aus heutiger Sicht erscheint dies inhaltlich und nomenklatorisch fragwürdig, da hier auf die Einlage eines Drainageröhrchens und dessen vermeintliche Infektionsgefahr Bezug genommen wird. Oder auch nachzulesen in der 14. Auflage des Duden von 1958, in dem „Dränage“ als „(Entwässerungs[anlage])“ erklärt wird, da der Terminus in seiner landwirtschaftlichen Verwendung sowohl den Vorgang der Entwässerung als auch die hierfür genutzte Entwässerungsanlage bezeichnen kann [16].

Aus oben genannten Gründen ist es daher auch präziser, von einem Paukenröhrchen zu sprechen, während „Paukendrainage“ eine verkürzte Bezeichnung für den Vorgang der Drainage, z. B. mithilfe eines Paukenröhrchens zum Abfließen (lassen) des Paukenergusses, z. B. nach einer Parazentese, wäre. Die primäre Funktion des Paukenröhrchens ist jedoch die Belüftung bzw. der Druckausgleich (vgl. engl. ventilation tube) [17, 18], was zu einem Rückbau der Becherzellen und mukösen Drüsen in der Paukenmukosa führt und nur sekundär eine Drainagefunktion hat, was so bereits von H. W. Pau propagiert und seinen Schülern so vermittelt wurde [19, 20]. In zeitgenössischen deutschsprachigen Lehrbüchern wird teilweise gänzlich auf den irreführenden Terminus „Paukendrainage“ verzichtet [21, 22] bzw. korrekt zwischen Paukendrainage und -röhrchen differenziert [23], wobei hier auch Inkonsistenzen auffallen [24, 25]. In letzter Konsequenz ist es somit auch aus inhaltlichen und orthografischen Gründen korrekt, im HNO-Alltag „PZ/PR“ (Parazentese/Paukenröhrchen) und nicht „PC/PD“ (Paracentese/Paukendrainage) zu gebrauchen [26, 27].

Es verwundert daher auch nicht, auf die Bitte nach einer „Abszessdrainage“ oder einer „Lymphdrainage“ keinen Gegenstand gereicht, sondern eine Operation bzw. eine physikalische Therapie und somit eine Prozedur zu erhalten. Auch kann ein „Redon“, benannt nach dem französischen Kieferchirurgen Henri Redon, nur im übertragenen Sinne in eine Op.-Wunde eingelegt werden [28].

All diese im Sprachgebrauch zu beobachtenden und mit der normierten Fachterminologie konfligierenden Unschärfen lassen sich aus sprachwissenschaftlicher Perspektive freilich erklären. Hier wirkt ein in der Alltagssprache weit verbreitetes, Metonymie genanntes semantisches Verfahren, nach dem etwa das Gefäß für den Inhalt („ein Glas trinken“), der Autor für sein Werk („Goethe lesen“) steht [29]. So kann nach dem Prinzip *totum pro parte* der gesamte Vorgang für das darin verwendete Instrument stehen. Alltagssprachlich verbreitete Formulierungen wie „eine Drainage legen“ werden so erklärlich. Hinzu kommt, dass das sog. Derivationssuffix *‑age*, vergleichbar mit dem deutschen *‑ung*, das zur Substantivierung von Verben (in diesem Fall *to drain*, ableiten) dient, generell semantisch unscharf ist und entsprechende Nominalisierungen oft mehrdeutig sind. Englisch marriage kann den Vorgang der Eheschließung ebenso bedeuten wie sein Ergebnis, den Ehestand. Als Überraschung kann die Handlung des Überraschens (nomen actionis) ebenso bezeichnet werden wie ein hierbei verwendeter Gegenstand wie in der Formulierung „eine Überraschung mitbringen“ (nomen instrumenti) [30, 31].

Wenn also in der alltäglichen Fachkommunikation und in Teilen auch in der Fachliteratur nicht hinreichend zwischen der Drainage als Vorgang und den hierbei verwendeten Gegenständen differenziert wird, so bewegt sich dies durchaus im Rahmen des deutschen Sprachsystems und seiner Wortbildungsprozeduren. Das sollte aber die Medizin als Wissenschaft nicht davon abhalten, ihre Termini zum Zwecke der Eindeutigkeit präzise zu fixieren.
